# Induction of the Stringent Response Underlies the Antimicrobial Action of Aliphatic Isothiocyanates

**DOI:** 10.3389/fmicb.2020.591802

**Published:** 2021-01-14

**Authors:** Dariusz Nowicki, Klaudyna Krause, Patrycja Szamborska, Adrianna Żukowska, Grzegorz M. Cech, Agnieszka Szalewska-Pałasz

**Affiliations:** Department of Bacterial Molecular Genetics, Faculty of Biology, University of Gdańsk, Gdańsk, Poland

**Keywords:** stringent response, (p)ppGpp, isothiocyanate, sulforaphane, enterohemorrhagic *Escherichia coli*

## Abstract

Bacterial resistance to known antibiotics comprises a serious threat to public health. Propagation of multidrug-resistant pathogenic strains is a reason for undertaking a search for new therapeutic strategies, based on newly developed chemical compounds and the agents present in nature. Moreover, antibiotic treatment of infections caused by enterotoxin toxin-bearing strain—enterohemorrhagic *Escherichia coli* (EHEC) is considered hazardous and controversial due to the possibility of induction of bacteriophage-encoded toxin production by the antibiotic-mediated stress. The important source of potentially beneficial compounds are secondary plant metabolites, isothiocyanates (ITC), and phytoncides from the Brassicaceae family. We reported previously that sulforaphane and phenethyl isothiocyanate, already known for their chemopreventive and anticancer features, exhibit significant antibacterial effects against various pathogenic bacteria. The mechanism of their action is based on the induction of the stringent response and accumulation of its alarmones, the guanosine penta- and tetraphosphate. In this process, the amino acid starvation path is employed via the RelA protein, however, the precise mechanism of amino acid limitation in the presence of ITCs is yet unknown. In this work, we asked whether ITCs could act synergistically with each other to increase the antibacterial effect. A set of aliphatic ITCs, such as iberin, iberverin, alyssin, erucin, sulforaphen, erysolin, and cheirolin was tested in combination with sulforaphane against *E. coli*. Our experiments show that all tested ITCs exhibit strong antimicrobial effect individually, and this effect involves the stringent response caused by induction of the amino acid starvation. Interestingly, excess of specific amino acids reversed the antimicrobial effects of ITCs, where the common amino acid for all tested compounds was glycine. The synergistic action observed for iberin, iberverin, and alyssin also led to accumulation of (p)ppGpp, and the minimal inhibitory concentration necessary for the antibacterial effect was four- to eightfold lower than for individual ITCs. Moreover, the unique mode of ITC action is responsible for inhibition of prophage induction and toxin production, in addition to growth inhibition of EHEC strains. Thus, the antimicrobial effect of plant secondary metabolites by the stringent response induction could be employed in potential therapeutic strategies.

## Introduction

Currently, the great challenges of modern medicine involve increasing resistance to known antimicrobial agents among pathogenic bacteria. This comprises a serious threat of spreading infections due to ineffective treatment leading even to pandemic outbreaks. The extensive and often unnecessary use of antibiotics eventually leads to the occurrence of resistance mechanisms due to bacterial evolution followed by spreading of antibiotic-resistance traits by vertical and, in particular, horizontal gene transfer within and in between bacterial species (Thomas and Nielsen, 2005). The amount of novel antibiotics yearly approved by the U.S. Food and Drug Administration (FDA) is decreasing, which is accompanied by increasing costs of developing new agents (Provenzani et al., 2020). Moreover, these antibiotics usually belong to the already known classes of antimicrobial agents and generally target specific groups of bacteria. This limits their possible usability in the control of infections caused by multidrug-resistant pathogens. Infections caused by antibiotic-resistant bacteria are the cause of at least 25,000 deaths in EU per year, and in addition, these infections are associated with increased costs of health care (Thorpe et al., 2018). This emerging problem creates a gap in the therapeutic agents that are available and leads to the need of extensive search for possible new antimicrobials, preferably differing in their mode of action from those already employed.

Natural products (especially plant-derived) are a promising source of potential compounds with antibacterial effects. As the secondary metabolites of the plants from the Brassicaceae family, isothiocyanates (ITC) gained particular attention due to their broad pro-health effects, including well-studied chemopreventive, anticancer, and antioxidant properties (Singh and Singh, 2012; Wiczk et al., 2012; Pawlik et al., 2013). However, the antibacterial effects of ITCs have not been sufficiently explored. In particular, understanding of ITC mode of antimicrobial action is still quite limited, with proposed underlying mechanisms including such effects as membrane disruption and cell lysis (Abreu et al., 2013; Patel et al., 2020). The unexpected and till recently unexplored mechanism of ITC antibacterial effect involves induction of bacterial stringent response. We have shown that ITCs, such as the widely studied sulforaphane (SFN), phenetyl ITC (PEITC), and benzyl ITC (BITC) inhibit bacterial growth of enterohemorrhagic *Escherichia coli* due to accumulation of the stringent response alarmone (p)ppGpp (Nowicki et al., 2014, 2016). These unusual nucleotides, the guanosine tetra- and pentaphosphate, are synthesized in *E. coli* by two enzymes. One is the RelA synthetase (*relA* gene product), responsive to amino acid starvation, and the other is the SpoT enzyme (*spoT* gene product), which is responsible for (p)ppGpp synthesis during other stresses and limitations, and is also responsible for ppGpp hydrolysis (Potrykus and Cashel, 2008). The ITC treatment interfered with amino acid metabolism, causing amino acid limitation, which in turn triggered RelA-mediated ppGpp accumulation (this effect was absent in the *relA* mutant strain). Moreover, excess of certain, but not all, amino acids, reversed this effect (Nowicki et al., 2014, 2016). Importantly, growth inhibition was accompanied by downregulation of the Shiga toxin synthesis. Expression of *stx* genes (coding for this toxin) is dependent on the lytic development of a lambdoid prophage, and it is the main factor responsible not only for virulence but for the life-threatening complications in humans, such as the hemolytic–uremic syndrome. Notably, the use of some common antibiotics to treat EHEC infections leads to the prophage induction and toxin synthesis, and therefore antibiotic therapy for this infection is disputable and high-risk. Thus, the development of novel methods to inhibit both, the bacterial growth and toxin synthesis, is an important aim in current biomedical research.

Phytoncides are a promising source of biologically active compounds, with various antibacterial activities. In terms of biological effects of ITCs, SNF is one of the best described (Houghton, 2019; Ruhee and Suzuki, 2020). Correlation between the chemical structure and antimicrobial effects of ITCs has been reported for some oral pathogens, where the degrees of these effects were compared for ITCs carrying various chemical groups (Ko et al., 2016). It was shown that the most potent antimicrobial effect was observed for indolyl ITC, followed by aromatic and aliphatic ITCs (Ko et al., 2016). The antimicrobial effects of some of these ITCs, namely, detoxification and anticancer activity, were already evaluated in the eukaryotic models (Munday and Munday, 2004; Milczarek et al., 2018). In this work, we asked what is an antibacterial effect of a set of sulforaphane analogs, also bearing aliphatic groups. Next, we tested the possible synergistic effect of various combinations of aliphatic ITCs and aimed to reveal the mechanism of their action. Our work presents evidence that the basis of the antimicrobial effect of ITCs, also when present in combinations, is accumulation of the stringent response alarmone, (p)ppGpp, induced through the amino acid starvation pathway.

## Materials and Methods

### Bacterial Strains and Growth Conditions

*E. coli* MG1655 and its *relA-* mutant (Xiao et al., 1991) were employed as standard laboratory strains. Clinical isolates of enterohemorrhagic *E. coli* O157:H7 were from our laboratory collection, EDL 933 and 86-24 Δ*stx*:GFP (Filipiak et al., 2020), and were used to evaluate ITC efficacy on human pathogens. Bacterial susceptibility tests were conducted at 37°C using the Mueller–Hinton (MH) broth (Sigma-Aldrich, Germany) or M9 (Sigma-Aldrich, Germany), with aeration and as indicated in the procedures’ description. The isothiocyanates were purchased from LKT Laboratories (United States).

### Estimation of Antimicrobial Effects

Antimicrobial activity of ITCs was assessed as described in Nowicki et al. (2019). The MIC values were estimated in accordance with the CLSI guideline M07-A9. Briefly, MICs were assessed by the twofold broth microdilution assay, and ITC concentrations ranged from 0.032 to 32 mM, and were later recalculated for mg/ml as an antibiotic concentration standard. Bacteria were grown in Mueller–Hinton (MH) or M9 medium on 96-well plates, with a final inoculum of 5 × 10^5^/ml. To determine the minimum bactericidal concentration (MBC), 100 μl of each culture was serially diluted (10^2^–10^5^) in saline, transferred onto MHA plates, and incubated at 37°C. Colony enumeration was carried out after 24 h. Cell suspensions without a given phytochemical were used as controls. The MBC was taken as the lowest concentration of phytochemicals at which no colony-forming units (CFU) were detected on solid medium. To assess amino acids’ impact on ITC antimicrobial effectiveness, 20 mM of each 20 amino acids was supplemented at time zero into M9 medium, and the MICs of ITCs were defined. The 20 mM concentration of amino acids was chosen to achieve their excess over the typical concentrations encountered by bacteria in the human intestines (Adibi and Mercer, 1973; Bener et al., 2006). Zone inhibition tests were assessed by the Kirby Bauer disc diffusion method, using 6 mm membrane discs (Biomaxima, Poland), with 30 *μ*g of chloramphenicol and 50 μg of a given ITC. Cell concentration of the tested microorganisms was adjusted to 0.5 McFarland turbidity standards, inoculated on MHA plates, and diameters of growth inhibition zones were measured after 20 h of incubation at 37°C. All presented results represent at least three independent experiments.

Time-kill assays were performed following the CLSI guidelines. Briefly, overnight bacterial cultures were suspended in MH medium and adjusted to an absorbance of approximately 10^6^ CFU/ml. Varying concentrations of the test compounds were added to the inoculum suspensions, with final concentrations corresponding to 1 x, 2 x, and 4 x MIC, and incubated at 37°C with aeration. Aliquots were removed from the inoculum culture after timed intervals of incubation (i.e., 0, 3, 6, 8, and 24 h), and serial 10-fold dilutions were prepared. Samples were plated on MH agar and incubated for 24 h at 37°C. Bacterial cell viability was determined by colony count. The assays were performed in triplicate. Data were analyzed as killing curves by plotting the log_10_ colony-forming unit per milliliter (CFU/ml) vs. time (hours), and the change in bacterial number was determined. The viable bacterial cell count for the time-kill end point determination, i.e., bactericidal activity, is defined as a reduction of ≥ 3 log_10_ CFU/ml relative to the initial inoculum, whereas bacteriostatic activity corresponds to < 3 log_10_ CFU/ml decrease relative to the initial inoculum.

### Fractional Inhibitory Concentration Index

The FIC index was used to estimate the synergy between different agents (Kang et al., 2011). Stock solutions and serial twofold dilutions of each drug (made to at least double the MIC) were prepared immediately prior to testing, as described (Bajaksouzian et al., 1996). A total of 50 μl of M9 medium (0.2% glucose) was distributed into each well of the microdilution plates. The first compound of the combination was serially diluted along the ordinate, while the second drug was diluted along the abscissa. An inoculum equal to a 0.5 McFarland turbidity standard was prepared in M9 (for SFN vs. ITC testing). Each microtiter well was inoculated with 100 μl of a bacterial inoculum of 5 × 10^5^ CFU/ml, and the plates were incubated at 37°C for 18–20 h under aerobic conditions. The resulting checkerboard contains each combination of two substances, with wells that contain the highest concentration of each compound at opposite corners, as was presented on isobolograms. The FIC index was calculated as follows:

$$F\,I\,C\,I=\left[\frac{M\,I\,C_{A\,\left(A+B\right)}}{M\,I\,C_{A\,\left(a\,l\,o\,n\,e\right)}}\right]+\left[\frac{M\,I\,C_{B\,\left(A+B\right)}}{M\,I\,C_{B\,\left(a\,l\,o\,n\,e\right)}}\right]$$

The FIC index was calculated on the basis of the abovementioned equation, in which the FIC index = X + Y, and the interactions are defined as: FIC index ≤ 0.5, synergy; 0.5 ≤ FICI ≤ 1.0, additive; 1.1 ≤ FICI ≤ 2.0, indifferent; and FICI > 2.0, antagonistic (Kang et al., 2011).

### Determination of (p)ppGpp Cellular Levels

The cellular levels of the alarmones, i.e., ppGpp and pppGpp, were measured basically as described (Mechold et al., 2002), with minor modifications as in Nowicki et al. (2014). Briefly, overnight bacterial culture grown in MOPS (4-morpholinepropanesulfonic acid) minimal medium was diluted in the same medium, but with a low phosphate concentration (0.4 mM), and then grown to A_600_ of 0.2. Then, bacteria were diluted (1:10) in the same medium with the addition of [^32^P]orthophosphoric acid (150 μCi/ml) and grown for at least two generations. Next, at time 0, ITCs at 1 × MIC or serine hydroxamate (SHX) at 1 mM were added to induce the stringent response by this amino acid analog. Bacterial samples, collected at specified time points, were lysed with formic acid (13 M) in three cycles of freeze–thaw procedure. After centrifugation, nucleotide extracts were separated by thin-layer chromatography, using PEI cellulose plates (Sigma-Aldrich, Germany) in 1.5 M potassium phosphate buffer (pH 3.4). The chromatograms were then analyzed with a Phosphorimager (Typhoon, GE Healthcare). QuantityOne software was used for densitometry analysis.

### Shiga-Toxin Production Analysis by Fluorescence Microscopy

Shiga toxin expression was monitored by GFP fluorescence as described in Nowicki et al. (2019). First, the overnight *E. coli* 86-24Δ*stx*:GFP culture was regrown (1:100 dilution) in fresh MH broth to OD = 0.2. Samples of bacterial cultures (1 ml) were withdrawn to 1.5 ml tubes and washed twice with PBS. Next, bacteria were resuspend in MH broth with or without ITCs (1x MIC). Mitomycin C (0.5 μg/ml) was used as the toxin synthesis inducer and was added to samples at time zero. Cell membranes and phenotypes were visualized by staining for 10 min with the SynaptoRed fluorescent dye (Sigma-Aldrich), at a final concentration of 5 μg/ml. To conduct vital staining and immobilize the culture samples, the thin pads of 1.5% agarose (dissolved in MH medium) were prepared then visualized using a Leica DMI4000B microscope fitted with a DFC365FX camera (Leica). The following Leica filter sets were used: N2.1 (for FM4-64), green fluorescent protein (GFP). Images were collected and processed using LAS AF 3.1 software (Leica).

### Phenotype Microarray Analysis

The phenomic analysis of the wild-type strain and *relA* mutant in comparison to strains treated with sulforaphane was carried out using Phenotype MicroArrays for Microbial Cells (Biolog). Duplicate runs of each of the strains were done and pairwise comparisons were created using OmniLog^®^ V.1.5 (Biolog, 178 United States). A reproducibility analysis was performed, and all strains passed the test. This analysis was done commercially by Biolog (Biolog Inc., United States).

Briefly, cellular response was measured for utilization of selected compounds, which led to changes in color development of tetrazolium violet. Here, we selected only plates—testing cells’ ability to utilize different nitrogen sources. Sulforaphane was added just before placing the inoculated fluids in the wells of each plate, to ensure there was the same SFN final concentration in each well (0.0625 mM; stock was made as 200 mM in 0.5% DMSO). The kinetic curves are the result of colorimetric measurements taken every 15 min for 48 h. Reproducibility analysis indicates the number of wells where the difference of average height between duplicate runs is above the threshold value—the “average height,” which is equivalent to the area under the curve divided by the number of reads (two). Pass/fail is determined by the number of such wells above the threshold value. The same procedure was done for wild-type strain and *relA* mutant. Those values are referred to as relative phenotype strength. Negative values refer to phenotype lost (upon SFN treatment), and positive values refer to phenotype gained (upon SFN treatment). Zero value means that the selected phenotype is not affected (changed) upon SFN treatment.

### Statistical Analyses

All experiments were performed independently in triplicates, and the data are presented as mean ± standard deviation. The significance of differences between mean values of two measured parameters was assessed by the *t*-test. Differences were considered significant when *P*-values were < 0.05.

## Results

### Sulforaphane Analogs Inhibit Bacterial Growth

**FIGURE 1:**
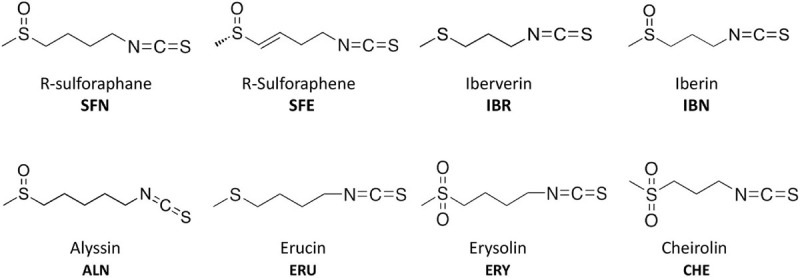
Chemical structure of isothiocyanates (ITCs) employed in this study.

**FIGURE 2:**
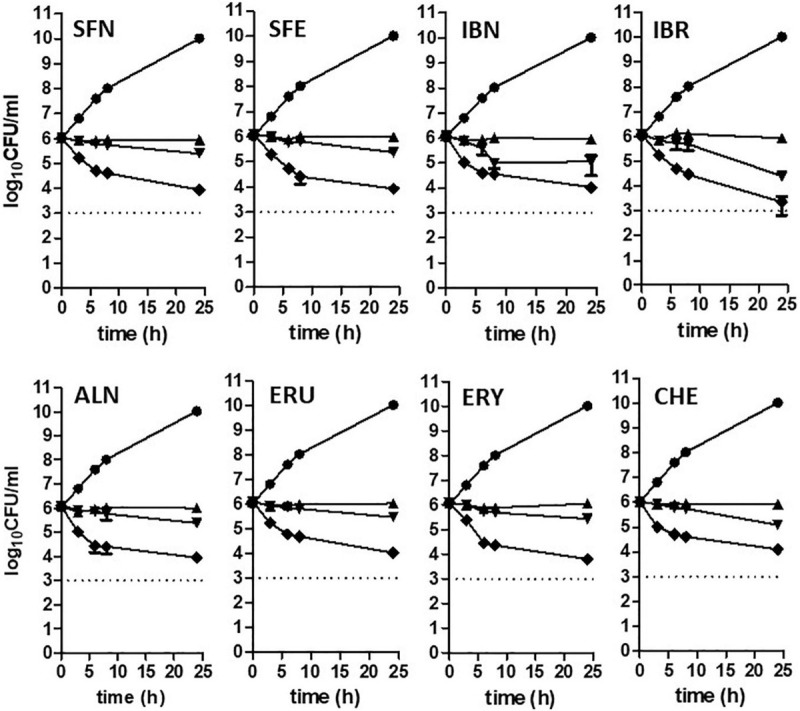
Time-kill kinetics at a range of concentrations of ITCs. *Escherichia coli* MG1655 culture was challenged with compounds at 1 x, 2 x, and 4 x MIC levels and compared to untreated control (triangle, reversed triangle, diamond, and circle, respectively). Bactericidal activity was defined as a reduction of 99.9% (≥ 3 log_10_) of the total number of CFU/ml in the original inoculum and marked as a dashed line on plots. Data are presented as mean and standard deviation of three independent replicates.

Isothiocyanates are a broad group of compounds derived from glucosinolates and produced after enzymatic cleavage by myrosinase in plant tissues. To elucidate their antibacterial mechanism of action, we focused here on those produced in nature though one pathway of a glucosinolate precursor, i.e., the aliphatic group of ITCs, which includes sulforaphane (SFN), sulforaphene (SFE), iberverin (IBR), iberin (IBN), alyssin (ALN), erucin (ERU), erysolin (ERY), and cheirolin (CHE) (Figure 1). We employed R-sulforaphane, which is naturally present in the plants (in contrary to the chemically synthesized mix of R and S sulforaphane) and its biological activity was reported as more potent than its S enantiomer (Abdull Razis et al., 2011). Initially, we evaluated the antimicrobial effect of these ITCs by the standard disc diffusion assay and microdilution method in order to characterize minimal inhibitory and bactericidal concentrations (Table 1). The screening evaluation of growth inhibition zones indicated that all tested compounds exhibit antimicrobial activity against *E. coli* MG1655 laboratory strain. Only ERU caused a lower inhibition of *E. coli* growth in the disc diffusion test (16.6 ± 0.3 mm), while all other ITCs showed ZI > 20 mm. The average inhibition zone of the negative control and the antibiotic chloramphenicol (used as a positive control) against the same target bacteria was 6.0 ± 0.0 mm (diameter of the disk) and 19.67 ± 1.4 mm, respectively. Further examination, employing microdilution assay in the MH medium and plating on solid MHA showed more variety in ITC effectiveness. Namely, among the SFN analogs, the MIC of only SFE was below 100 mg/L (87.7), while MIC values for IBR, IBN, ALN, ERU, ERY, and CHE were approximately twofold higher (147.3, 163.3 191.3, 161.29, 125, and 125 mg/L, respectively) (Table 1). SFE showed a potent bactericidal action, similar to SNF, expressed in MBC equal to MI concentration (87.7, 88.6 mg/L, respectively). The MBC of other ITCs were two to fourfold higher than their MIC. Moreover, we demonstrated that the composition of culture medium had an impact on antibacterial effectivity of the tested ITCs. MIC values obtained in M9 medium (+0.2% glucose) were ∼10-fold lower in comparison to the rich MH broth. In the absence of amino acids and peptides in the culture media, SFN, SFE, and IBN exhibited the strongest action (MIC of 5.5, 5.5, 5.1 mg/ml), while for ERU, we observed approximately fourfold MIC decrease (40.3 mg/l). Finally, the MBC assessed in poor nutrient conditions were twofold higher than MIC for ERY and CHE and were equal with MIC for the rest of the tested compounds. The time-kill assay performed with the tested compounds confirmed their bacteriostatic effect (Figure 2). Thus, we can conclude that the effectiveness of the antimicrobial effect is correlated with both the type of ITC used and the bacterial growth conditions.

**TABLE 1:** Antimicrobial activity of ITCs.

**Broth**	**Mueller–Hinton**			**M9**	
**ITC:**	**MIC mg/L (mM)**	**MBC mg/L (mM)**	**ZI mm**	**MIC mg/L (mM)**	**MBC mg/L (mM)**
SFN	88.6 (0.5)	88.6 (0.5)	22.3 ± 1.0	5.5 (0.03)	5.5 (0.03)
SFE	87.7 (0.5)	87.7 (0.5)	25.8 ± 0.3	5.5 (0.03)	5.5 (0.03)
IBR	147.3 (1.0)	294.6 (2.0)	23.3 ± 0.6	9.2 (0.06)	9.2 (0.06)
IBN	163.3 (1.0)	326.6 (2.0)	24.1 ± 1.6	5.1 (0.03)	5.1 (0.03)
ALN	191.3 (1.0)	382.6 (2.0)	20.8 ± 0.3	11.9 (0.06)	11.9 (0.06)
ERU	161.3 (1.0)	645.2 (4.0)	16.6 ± 0.3	40.3 (0.25)	40.3 (0.25)
ERY	125 (0.65)	500 (2.6)	25.1 ± 1.0	14.8 (0.08)	29.6 (0.15)
CHE	125 (0.7)	500 (2.8)	24.9 ± 1.0	14.8 (0.08)	29.6 (0.17)

*Antimicrobial potential against E. coli MG1655 expressed as a minimal inhibitory concentration (MIC) and minimal bactericidal concentration (MBC), as well as zone inhibition (ZI) diameter.*

### The Stringent Response Induction Underlies Antibacterial Action of Aliphatic Isothiocyanates

**FIGURE 3:**
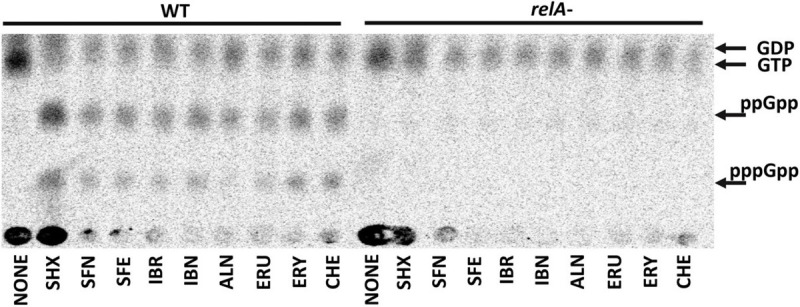
The effect of isothiocyanates on the (p)ppGpp alarmone accumulation in *E. coli*. Bacteria were grown overnight on LB agar plates at 30°C, then collected and washed with PBS buffer, concentrated, and resuspended in low phosphate MOPS labeling medium at OD_600_ = 0.2 density. Cells were labeled with 5 μCi/ml ^32^P for 20 min. (p)ppGpp synthesis was induced with 1 mg/ml of serine hydroxamate (SHX) for positive control; various isothiocyanates were used at 1 x MIC concentration for 20 min. Samples were spotted on PEI cellulose TLC plates, developed in 1.5 M potassium phosphate buffer and visualized with a Phosphoimager. The positions of guanosine nucleotides (GDP, GTP, ppGpp, and pppGpp) are indicated by arrows.

Following our previous research (Nowicki et al., 2016, 2019), we tested selected SFN analogs for their potential to elevate the (p)ppGpp alarmones level to reveal the effect of these ITCs on cellular stress response. The results presented here (Figure 3), strongly indicate that ITC-mediated bacterial growth inhibition is caused by global metabolism alterations accompanied by enhanced production of the stress alarmones, which is in agreement with the effect reported for SFN. Based on the previous results (Nowicki et al., 2014, 2016), where the involvement of the amino acid starvation pathway was shown as a mechanism responsible for ITC-mediated (p)ppGpp accumulation, we then employed an *E. coli* strain devoid of the *relA* gene. As we did not observe (p)ppGpp accumulation in ITC-treated *relA* mutant, we could conclude that these compounds act by inducing amino acid starvation. However, the actual direct effect of ITCs has an impact on *relA* mutant strain as well, since their sensitivity to the tested compounds is at least at the same level or higher in comparison to the wild-type bacteria (Supplementary Table S1). Therefore, in order to explain this phenomenon, we used an excess of individual free L-amino acids (20 mM) to check whether it affects ITC action. In this analysis, we showed that the antimicrobial action of SFN analogs was impaired by specific amino acids (Table 2). The highest reversal effect was observed for SFN and SFE in the presence of glycine and methionine; for other ITCs, glycine was able to increase MIC up to about eightfold. Moreover, some moderate impact of other amino acids was also observed for cultures supplemented with tyrosine, glutamine, cysteine, tryptophan, alanine, and phenylalanine (≥ fourfold MIC increase), while at the same time, none of the amino acids could stimulate ITC antibacterial potential.

**TABLE 2:** The impact of particular amino acids which can alter ITC antimicrobial action against *E. coli* MG1655.

**ITC:**	**8 x MIC**	**4 x MIC**
SFN	Gly, Met	Tyr, Gln, Trp, Cys, Arg
SFE	Gly, Met	Tyr, Gln, Trp, Phe, Ala
IBR	Gly	Met, Tyr, Gln, Phe
IBN	Gly	Met, Tyr, Gln, Trp, Phe, Ala
ALN	Gly	Met, Tyr, Gln, Phe, Ala
ERU	Gly	Met, Tyr, Gln, Ala
ERY	Gly	Met
CHE	Gly	Met, Asn

*The amino acids were assigned into rows according to the strength of their activity, expressed as increased fold change of MIC value for specific ITC (four and eight times, respectively).*

### The Synergistic Antimicrobial Effect of Isothiocyanate Combinations Results From the Stringent Response Induction

**FIGURE 4:**
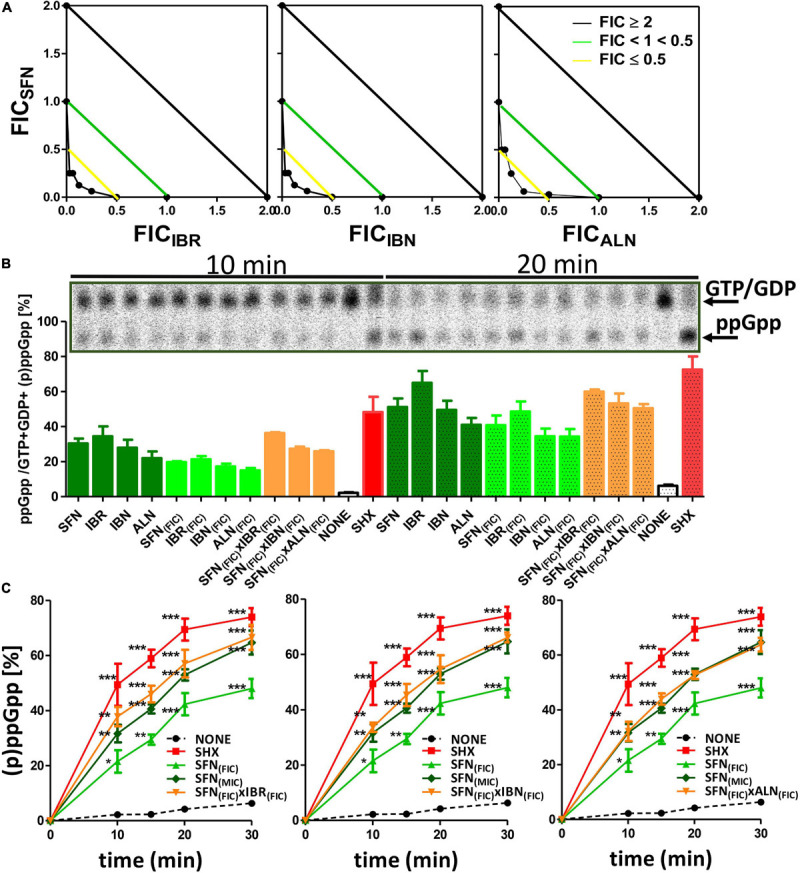
Analysis of synergistic interactions between IBR, IBN, ALN, and SFN. **(A)** Synergistic effects of sulforaphane in mixtures with iberin, iberverin, and alyssin are represented on isobolograms. Estimated FICI values are presented for combinations of SFN with other ITCs. A checkerboard technique was employed to delineate the Fractional Inhibitory Concentration Index (FICI). The treated cultures were screened for visual growth in a microplate reader. The FICI were then calculated as described in section “Materials and Methods” (FICI ≤ 0.5, synergy; 0.5 ≤ FICI ≤ 1.0, additivity; 1.1 ≤ FICI ≤ 2.0, indifference; FICI ≥ 2.0, antagonism) **(B)** ppGpp alarmone accumulation under treatment with synergistic combinations of SFN + ITCs. **(C)** The kinetics of relative ppGpp accumulation in treated cells. Relative ppGpp levels were assessed by densitometry using the QuantityOne Software. The ^33^P incorporation method was used to evaluate stringent response induction like described previously. (p)ppGpp synthesis was induced with 1 mg/ml of serine hydroxamate (SHX) for positive control; various isothiocyanates were used in 1x MIC or FIC concentration in combination for 10, 15, 20, and 30 min. Samples were spotted on PEI cellulose TLC plates, developed in 1.5 M potassium phosphate buffer and visualized with a Phosphoimager. The results are from at least three independent experiments. The pooled (p)ppGpp and GTP amounts were taken as 100%. The statistical significance of differences in (p)ppGpp amount compared to its basal level of samples at the corresponding time of non-treated control was determined by *t*-test (*p ≤ 0.05, **p ≤ 0.01, and ***p ≤ 0.001) as indicated above or below plots.

Any effect exerted by an individual compound may vary from the one observed for the combination of various chemicals. This also holds true for the antibacterial agents. Knowing the potential of SFN and its analogs to inhibit bacterial growth, we asked whether the simultaneous action of two ITCs may give a synergistic outcome. For this, we combined SFN with individual ITCs tested previously, and we assessed bacterial growth inhibition by checkerboard titration. In this way, the fractional inhibitory concentration (FIC) index was established for each combination (Table 3). The synergistic effects of SFN and IBR, SNF and IBN, and SFN and ALN led to the reduction in MIC for these compounds by four- to eightfold, with the FIC index of ≤ 0.5 indicating synergy. The combinations of SNF with IBR, and IBN and ALN, resulted in the additive effect of the antimicrobial actions (the FIC index value was ≥ 0.5, Figure 4A), while others gave additive effects (Supplementary Figure S1A). Interestingly, the antibacterial effects of combinations of these ITCs, which acted synergistically with SFN (IBR, IBN, ALN), were synergistic only for the iberin–iberverin mixture, while for the allysin in combination with iberin and iberverin, the effect was additive (Supplementary Figure S1B). Thus, these data indicate that some of the combinations of SFN with its analogs can give a synergistic antibacterial effect.

**TABLE 3:** Estimated FICI values for combinations of ITCs.

**ITC combination (A × B)**	**Combined MIC values (mg/L)**	**FIC_*A*_ + FIC_*B*_**	**FICI**
SFN + SFE	1.39 + 2.77	1/4 + 1/2	0.75 ≥ 0.5 ADD
SFN + IBR	0.69 + 1.15	1/8 + 1/8	0.25 ≤ 0.5 SYN
SFN + IBN	1.39 + 0.64	1/4 + 1/8	0.375 ≤ 0.5 SYN
SFN + ALN	1.39 + 1.5	1/4 + 1/8	0.375 ≤ 0.5 SYN
SFN + ERU	1.39 + 20.2	1/4 + 1/2	0.75 ≥ 0.5 ADD
SFN + ERY	1.39 + 7.4	1/4 + 1/2	0.75 ≥ 0.5 ADD
SFN + CHE	0.69 + 7.4	1/8 + 1/2	0.625 ≥ 0.5 ADD
IBR + IBN	1.15 + 0.64	1/8 + 1/8	0.25 ≤ 0.5 SYN
IBR + ALN	2.3 + 5.95	1/4 + 1/2	0.75 ≥ 0.5 ADD
ALN + IBN	1.5 + 2.55	1/8 + 1/2	0.625 ≥ 0.5 ADD

*FICI results ≥ 0.5 were considered as additive interactions and ≤ 0.5 as synergistic.*

**FIGURE 5:**
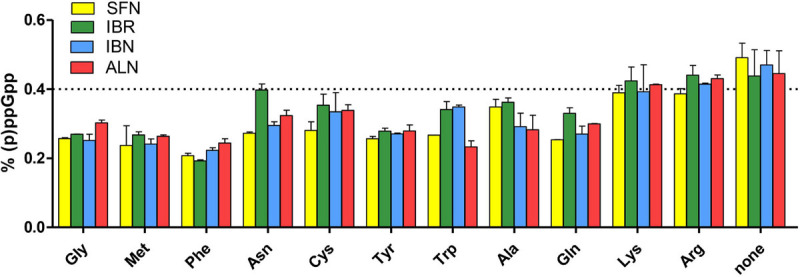
The effect of specific amino acids on the stringent alarmone (p)ppGpp synthesis during SFN, IBR, IBN, and ALN treatment of *E. coli* MG1655. Relative (p)ppGpp accumulation after supplementation with specific amino acids in cultures treated with SFN (yellow), IBR (green), IBN (blue), and ALN (green). The assessment of intracellular level of (p)ppGpp alarmones was determined by [^32^P]orthophosphoric acid incorporation and developed by TLC on PEI cellulose plates, followed by densitometry. The level of (p)ppGpp represents % of a sum of all G nucleotides visualized on TLC plate. The dotted line represents the mean level of alarmone induction in SFN, IBR, IBN, and ALN treated cells without amino acid supplementation (control). The results are from at least three independent experiments.

As the bacterial growth inhibition caused by the tested ITCs is straightforwardly related to the induction of the stringent response (Figure 3), we assessed the accumulation of (p)ppGpp alarmones upon SNF treatment in combination with those ITCs, which showed the synergistic effect (namely, IBR, IBN, and ALN). The level of accumulated (p)ppGpp in the presence of SFN/ITC combination at the FIC concentrations was comparable to this observed for MIC concentrations of individual compounds (Figure 4B). This indicates that the combination of several fold lower concentration of ITCs (FICs) gives a similar effect for the stringent response induction as the much higher amounts of individual ITCs (MICs). Interestingly, the kinetics of (p)ppGpp accumulation upon these various treatments showed that the level of the stringent response induction, in terms of amount and time, was similar for the MIC values of SNF and combination of FIC values of SNF and either of three ITC showing synergistic effect (Figure 4C). Namely, the levels of accumulated (p)ppGpp expressed as % of pooled guanosine nucleotides were 64.7 [SFN_(MIC__)_], 66.6 [SFN_(FIC)_ + IBR_(FIC__)_], 66.1 [SFN_(FIC)_ + IBN_(FIC__)_], and 63.7 [SFN_(FIC)_ + ALN_(FIC__)_] at the end point of the experiment (30 min). Thus, the stress caused by SNF/ITC action is sufficient to achieve cellular (p)ppGpp concentrations inhibiting bacterial growth. Furthermore, we asked how the amino acids Met and Gly, which effectively counteract bacterial growth inhibition, can impair the (p)ppGpp accumulation induced by SFN, IBR, IBN, and ALN. The observed estimated levels of alarmones in the Met and Gly treated cells were significantly reduced (by ∼40%) in comparison to cells treated with an ITC alone (*p* < 0.05) (Figure 5). Nevertheless, we also found that Phe and Thr supplementation also acts negatively on the alarmone accumulation, while the impact of other amino acids was moderate and varied depending on a particular ITC.

### The Antibacterial Potential of Sulforaphane Analogs Against Enterohemorrhagic *Escherichia coli* Strains

**FIGURE 6:**
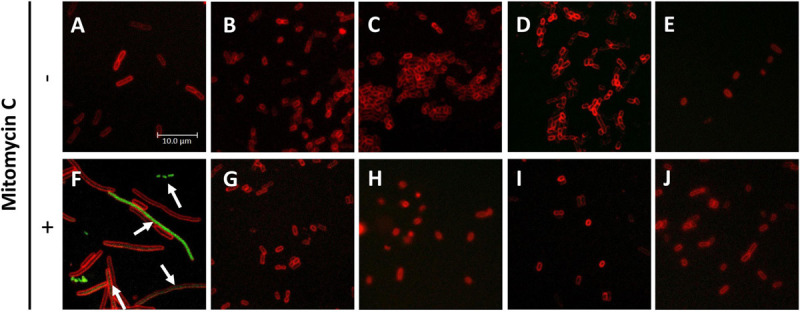
Inhibition of *stx*_*p*_ activity under ITC treatment of *E. coli* 86-24 O157:H7 strain. *E. coli* 86-24 O157:H7 Δ*stx*:GFP was cultivated for 3 h in the presence of **(A)** not-treated control **(B,G)**, SFN added at MIC value, **(C,H)** SFN and IBR at FIC values, **(D,I)** SFN and IBN added at FIC values, **(E,J)** SFN and ALN at FIC values. **(F–J)** Mitomycin C was added as the toxin production inducer. Bacteria were then stained with SynaptoRed to visualize membranes; GFP synthesis was analyzed by fluorescence microscopy. The activity of stx_*p*_:GFP is marked by arrows. Pictures present merged green and red channels; the scale bar is valid for all panels.

The actual aim of the studies on antimicrobial agents is to assess their effectiveness against pathogenic strains. Therefore, we tested the antibacterial effect of SNF and its analogs on the enterohemorrhagic *E. coli* strains: EDL 933W and 86–24, carrying Shiga toxin-converting prophages. Interestingly, their growth inhibition was at least at the level observed for the wild-type *E. coli* (Table 3) for SFN, IBN, ALN, and CHE, while for SFE, IBR, ERU, and ERY, the MIC values were even lower. These results indicate that pathogenic *E. coli* strains are sensitive to the aliphatic ITCs. Because of the specific virulence of EHEC strains, the therapeutic agents should not only stop bacterial growth but, more importantly, inhibit lytic development of *stx*-bearing prophages, preventing Shiga toxin production. The *E. coli* 86–24 Δ*stx*:GFP strain with GFP gene replacing the *stx* gene was used to monitor the ITC effect on prophage induction. The GFP gene expression was mediated by *stx* promoter. In this experiment, mitomycin (0.5 μg/ml) was used to induce phage development (Otsuji et al., 1959), which resulted in the impairment of cell division and subsequent filamentation (Figure 6F). At the same time, GFP synthesis was visible as a result of the expression of prophage genes, including the region with the *stx* gene, during phage lytic development (Figure 6F). The ITC treatment alleviated both effects of mitomycin: the filamentation phenotype was reversed and GFP synthesis was at an undetectable level; this effect was observed for SNF alone, and for SNF in combination with IBR, IBN, and ALN (Figures 6G–J). ITC treatment at FIC concentrations (analogous to the growth inhibition experiments described above) in the absence of mitomycin did not induce prophage induction and *stx* promoter activity (monitored by GFP production) (Figures 6B–E), although the stress conditions caused by ITC resulted in decreased cell size (compare Figure 6A with Figures 6B–E). These results confirmed that ITCs, acting individually or synergistically, decrease growth of EHEC strains without induction of the lambdoid prophage lytic development.

### Nitrogen Source Utilization Upon Sulforaphane Treatment

**FIGURE 7:**
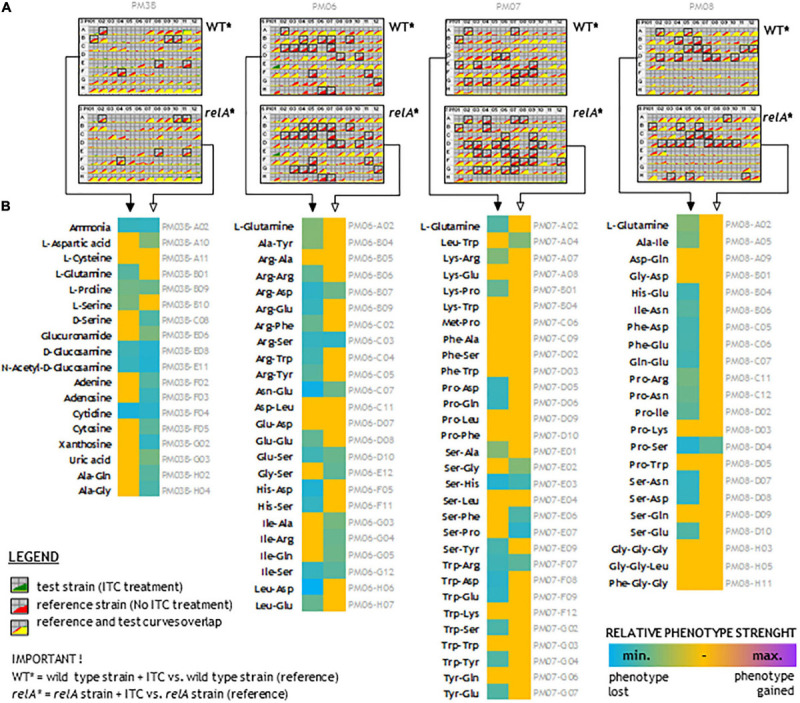
N-source utilization upon sulforaphane treatment. **(A)** Kinetic curves of tetrazolium violet (TV) color development upon 48 h of sulforaphane (ITC) treatment. Each plate shows comparison of treated and untreated strains, as indicated. The yellow color reflects the situation where the tested treated strain utilizes nitrogen-rich compounds at the same level as untreated corresponding strain. The red color reflects reduced metabolic flow in the treated strains (most cases). Black frames indicate scored changes in kinetics that passed the reproducibility test. **(B)** The effect of ITC treatment on the wild-type and *relA* mutant strains. The more turquoise the color, the more ITC treatment suppresses utilization of a given nitrogen compound. Full arrows point to the wild-type strains and open arrows to the *relA* mutants. Descriptions in gray refer to the nitrogen compound plate position.

To understand the wide effect of SFN on bacteria, we performed a downstream analysis of bacterial phenotypes due to nitrogen source utilization upon sulforaphane treatment. Many nitrogen-rich (N-rich) compounds that can be transported into a cell and metabolized to produce NADH, will generate a redox potential and flow of electrons to reduce a tetrazolium violet (TV), thereby developing the color change. The more rapid this metabolic flow is, the more quickly the color is formed. The metabolic flow is supressed or unchanged by adding sulforaphane. Figure 7A presents the TV reduction kinetics data of wild-type strain and *relA* mutant treated with SFN in comparison to untreated strains (the controls). The curves show the time course (horizontal axis) of the amount of color formed from tetrazolium dye reduction (vertical axis) in each of the 96 wells. Reference (control) is shown in red and Test (compound added) is shown in green (Figure 7A). If both curves overlap (the yellow area), it gives an information that there is no difference between the strain tested and not tested with SFN, in the presence of a particular N-source. If the effect of ITC treatment reduces the metabolic flow, the red curves occur (many cases). Measurements that pass the reproducibility test are marked with black box (Figure 7A).

The data presented for this experiment strongly suggest that despite SFN treatment, many N-source are metabolized on the same level in the untreated controls. The data show that upon SFN treatment, many amino acids are poorly metabolized as a sole N-source, in both analyzed strains. However, utilization of several N-compounds is not affected upon ITC treatment. Therefore, we have analyzed if are there any correlations between the strength of the phenotype and the physicochemical properties of dipeptides (and some tripeptides). We have employed several parameters to the analysis, such us molecular weight, extinction coefficient, iso-electric point, net charge at pH 7, estimated solubility, and hydrophilicity. We found that for a wild-type strain treated with sulforaphane, there is moderate (but significant) negative correlation between estimated solubility of the peptide and the strength of phenotype. Also, we found that there is a low negative correlation between hydrophilicity and the strength of a given phenotype (Supplementary Table S2).

## Discussion

In this work, we showed that all tested aliphatic ITCs specifically induce stringent control through the amino acid pathway, controlled by RelA. Moreover, the alarmones’ accumulation increases in time (Figure 4C), which suggests an ongoing stress response. Thus, the antibacterial effect of the ITC treatment is related to the stringent response and to the factors triggering induction of (p)ppGpp accumulation. These factors lead to amino acid starvation, in both, the wild-type and (p)ppGpp-devoid strains; however, in the wt bacteria, the rapid induction of the stringent response results in the subsequent downregulation of the main metabolic processes. Thus, this specific effect of ITCs is responsible for growth arrest, rather than a consequence of bacterial cell disintegration as proposed by others (Borges et al., 2015; Dufour et al., 2015; Romeo et al., 2018). The alterations in bacterial metabolism, resulting from an indirect effect of (p)pGpp accumulation and/or the actual direct action of ITCs related to amino acid limitation leading to downregulation of protein synthesis, can eventually lead to cellular membrane disruption and cell death. Thus, ITCs can affect both, the wild type and (p)ppGpp-deficient bacteria, which is strongly supported by the sensitivity of *relA* mutant strain to ITCs (Supplementary Table S1). The importance of the stringent response induction in the wild-type strain lies in its effect on Shiga toxin production by EHEC strains, as we reported previously (Nowicki et al., 2014, 2016).

The chemical nature of aliphatic ITCs as particularly reactive compounds makes them a subject to nucleophilic attack at the electron-lacking central carbon atom (Wu et al., 2009). In the presence of thiol molecules, this carbon atom is attacked, and dithiocarbamates are formed (Shibata et al., 2011). It has been shown that inhibition of the sulfhydryl enzymes may play a role in the antimicrobial action of ITCs (Luciano and Holley, 2009). Nevertheless, amino acid deficiency is the first observed effect under ITCs treatment, as we showed here and in our previous work (Nowicki et al., 2014, 2016).

The involvement of ITC-mediated effect in the amino acid availability has been already shown for some ITCs (Nowicki et al., 2013, 2016). The potential interaction of ITCs with amino acids was concluded from the observation that excess of specific amino acids reversed antibacterial action of ITCs. Interestingly, the observed effects were related to the ITCs structure. Selected ITCs, among them those with aliphatic, branched chained, and aromatic groups, exhibit different specificity toward interaction with various amino acids. There were some amino acids common for specific ITCs, such as glycine for SFN, benzyl ITC, and allyl ITC, arginine for SFN, phenyl ITC, and IPRITC, phenylalanine for BITC and AITC, or lysine for PITC and IPRITC. We also noticed that glycine was the most effective in growth arrest recovery (Nowicki et al., 2013, 2016). Here, we aimed to elucidate the possible interactions of amino acids with analogs of SNF. We found here that all aliphatic ITCs tested, seem to effectively interact with Gly and Met, and specifically with other amino acids with different strengths (Table 2). It is known that the electron-deficient central carbon of ITCs is susceptible to attack from amino groups, forming thiourea derivatives (Wu et al., 2009). The electrophilic action of AITC was observed while studying reactions between ITCs and proteins (Kawakishi and Kaneko, 1985, 1987). The ability of AITC to initiate disulfide bond oxidative cleavage in cysteine moieties and to react with free amino groups of lysine and arginine was identified (Weerawatanakorn et al., 2015). The interaction of isothiocyanates with cysteine was suggested in their interaction with tubulin (Xiao et al., 2012). Nevertheless, there is no evidence for the microbial proteins as specific targets. Therefore, it is unclear if the same effect would occur *in vivo*. Cejpek and colleagues also postulate that *in vitro*, there are interactions with free amino acids and short peptides (Cejpek et al., 2000). The binding of sulforaphane to a defined peptide sequence was found to be a basis of its interaction with the Hsp90 protein (Li et al., 2012), while the molecular docking modeling indicated the direct interaction of SFN with Asn in NAD(P)H:quinone oxidoreductase (Mazur et al., 2010). To challenge the phenomenon of ITC interaction with amino acids *in vivo*, we extended our investigation by BIOLOG phenotypic arrays analysis (Figure 7) for SFN-treated cells employing wt and *relA* strains. Cells were treated with 1/4 MIC value to provoke amino acid starvation without complete shutdown of bacterial growth. This study showed that there are specific interactions where di- and three- peptides and other N-sources can effectively restore bacterial metabolism in the presence of SFN. Specifically, a broad group of short peptides, namely, those containing Gly, Met, Phe, Gln, Trp, and Ala can affect the SFN antimicrobial potential. We even noticed a higher ability to utilize the peptides as a sole N-source in the *relA* strain. We assumed it is a consequence of impaired (p)ppGpp synthesis under amino acid starvation conditions, while due to increased alarmone level in the wt strain, the metabolism and other cellular processes remained altered and generally downregulated upon SFN treatment. However, we do not see any statistically significant correlation between any specific peptide features and the strength of phenotype for the *relA* mutant treated with SFN (Supplementary Table S2). There is no clear relationship between the physiochemical properties of N-course compounds and sulforaphane effect. Thus, the molecular mechanism of these interactions remains yet to be solved.

In the search for novel antimicrobials, a possible synergy between compounds is often considered. The phenomenon of synergy occurs when the effect of two compounds in the mixture exceeds the sum of their individual effects. Thus, such interaction results in the increased effectiveness of a given chemical mixture. Importantly, the synergistic effect requires lower concentrations of compounds for comparison with their individual action and decreasing the effective doses usually results in reduction of potential side effects. The synergy of the two components is utilized in, e.g., antibacterial therapy (Kang et al., 2011; Brooks and Brooks, 2014), with the example of a widely used combination of beta-lactam antibiotics and inhibitors of β-lactamases (Neu, 1987). Thus, in fighting antibiotic-resistant pathogens, the important part is not only the finding of novel compounds but also assessment of their effects with the already known agents. The mechanisms underlying the synergistic effects of various compounds involve interactions with different cellular targets, increasing bioavailability and pharmacokinetics of the compounds or activating effectiveness of an otherwise inactive compound (Wagner and Ulrich-Merzenich, 2009). For the antibacterial effects, the synergistic combinations decrease or delay the spread of bacterial resistance to one or both agents (Korcsmáros et al., 2007). For isothiocyanates, the main mechanism of their antibacterial action involves the stringent response induction, which is triggered by the amino acid starvation, as we have shown previously for PEITC, BITC, and SFN (Nowicki et al., 2014, 2016) and, in this work, for the sulforaphane analogs. The synergy in the situation of the same cellular target would seem difficult to envision; however, the effect of amino acid starvation can be attained by depletion of various amino acids. Indeed, the reversal of ITC effects was observed in the presence of various amino acids (Table 2), specific for each ITC, indicating possible divergent interaction with amino acids. This effect may involve amino acid transport system, which could be directly or indirectly blocked by ITCs. Therefore, the excess of amino acids would diminish the ITC impact. A possible effect on the aminoacyl tRNA synthetases can be also taken into consideration, as the lack of the specific aminoacyl tRNA would block the ribosomes and activate RelA. The available data on the ITC binding to amino acids and proteins, together with our results on the specificity of amino acid-mediated reversal of ITC effect, suggest that the interaction of isothiocyanates with amino acids (free or present in the proteins) is the basis of pleiotropic effects of ITCs.

The observed effects of individual ITCs or their combinations on the model and pathogenic strains of *E. coli* are underlined by the stringent response induction as a main mechanism of their antimicrobial properties. An antibacterial effect of (p)ppGpp accumulation is a very rare mechanism of an antibiotic mode of action. Generally, (p)ppGpp increases bacterial virulence by regulating the expression of pathogenicity island genes, promoting antibiotic resistance (Strugeon et al., 2016) and supporting occurrence of persister cells (Svenningsen et al., 2019) as reported recently, due to ribosome dimerization (Song and Wood, 2020). Thus, studies carried out so far usually focused on developing inhibitors of (p)ppGpp synthesis, e.g., by targeting enzymes responsible for (p)ppGpp synthesis (Wexselblatt et al., 2010, 2012, 2013) or leading to degradation of the alarmone molecules (de la Fuente-Núñez et al., 2014, 2015).

Therefore, the use of ITCs as antibacterial agents can be advantageous, as there are very few examples of bacterial resistance to these compounds, as reported for *Pseudomonas sax* genes related overcoming of host defense mechanisms in *Arabidopsis* (Fan et al., 2011). The bactericidal effect of ITC shown in time-kill assay confirms the antimicrobial effect of these compounds (Figure 2). The occurrence of persister cells upon ITC treatment was not reported so far; however, it cannot be excluded because of the increase in (p)ppGpp level, so this problem requires further studies. Our data show promising antibacterial effects of sulforaphane analogs against EHEC strains (Table 4). Taking into consideration that, on one hand, these ITCs are a part of normal diet, and their safety for humans is widely investigated for their chemoprevention properties, and on the other hand, the antibiotic treatment of EHEC infections is very limited, this could open broad opportunities to include ITCs as a potential therapeutic strategy based on the stringent response-mediated antimicrobial action.

**TABLE 4:** The determined ITC susceptibility of *E. coli* O157:H7 strains.

***E. coli* O157:H7**	**MIC mg/L (mM)**	
**ITC:**	**EDL 933W**	**EDL 933W**
SFN	88.6 (0.5)	88.6 (0.5)
SFE	43.9 (0.25)	43.9 (0.25)
IBR	73.7 (0.5)	73.7 (0.5)
IBN	163.3 (1.0)	163.3 (1.0)
ALN	191.3 (1.0)	191.3 (1.0)
ERU	80.6 (0.5)	80.6 (0.5)
ERY	62.5 (0.32)	62.5 (0.32)
CHE	125 (0.7)	125 (0.7)

## Data Availability Statement

The raw data supporting the conclusions of this article will be made available by the authors, without undue reservation, to any qualified researcher.

## Author Contributions

DN and AS-P conceived the study and designed the experimental procedures. DN, KK, PS, AŻ, and GC carried out the experiments. DN, AS-P, and GC analyzed the data and wrote the manuscript. AS-P supervised the project. All authors approved the final manuscript.

## Conflict of Interest

The authors declare that the research was conducted in the absence of any commercial or financial relationships that could be construed as a potential conflict of interest.
