# Supplementary material for: Induction of the Stringent Response Underlies the Antimicrobial Action of Aliphatic Isothiocyanates
Source: Front Microbiol. 2021 Jan 14;11:591802. doi: 10.3389/fmicb.2020.591802 (PMC7874123; doi:10.3389/fmicb.2020.591802)
Supplement: Supplementary file 1 [file Table_1.DOCX]

Supplementary Material

1. **Interactions between SFN analogues**


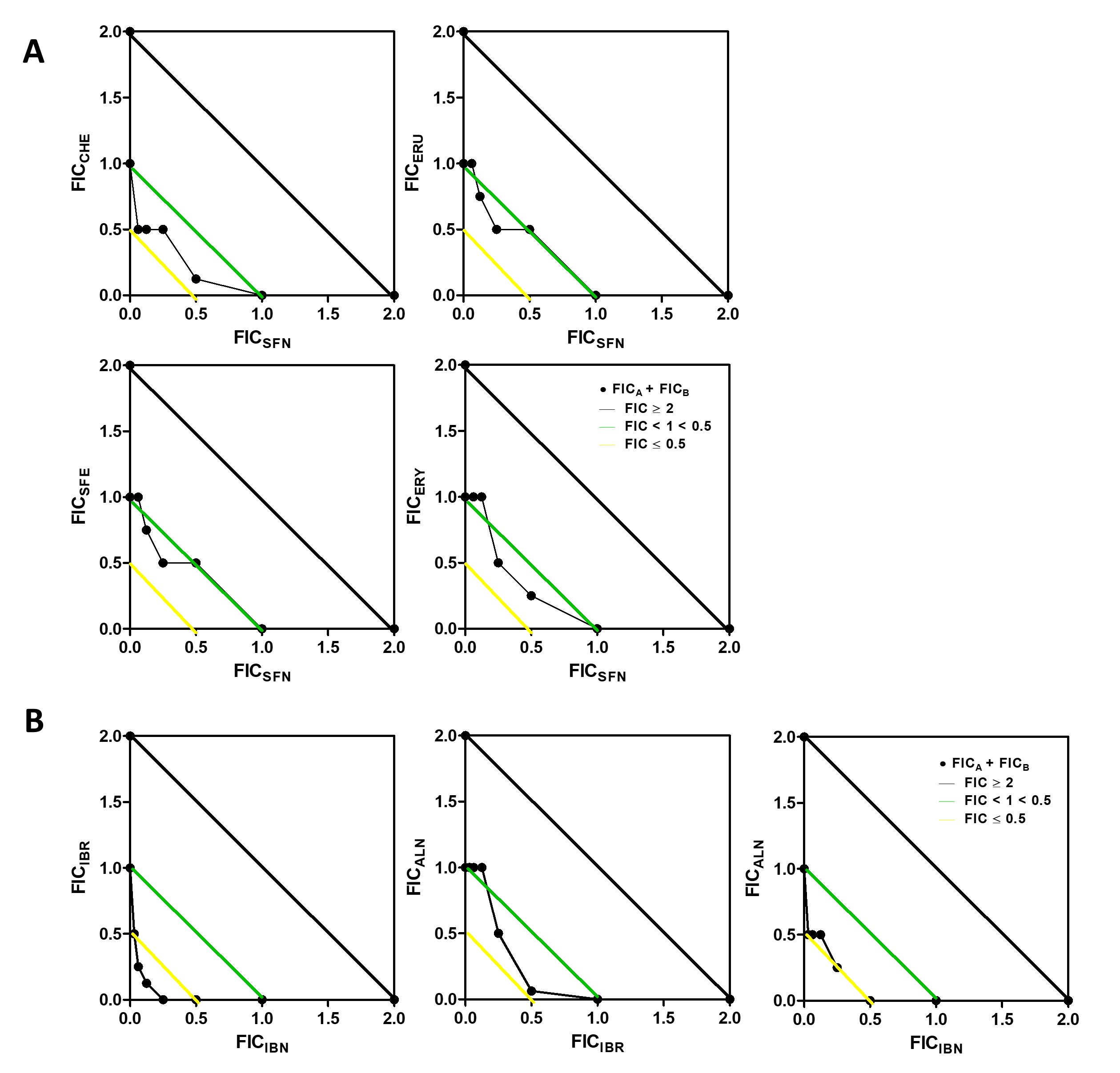


**Supplementary Figure 1.** **Analysis of antibacterial interactions of ITC.**

(A) The effects of sulforaphane in mixtures with sulforaphane, erucin, erusolin, cheireolin are expressed on isobolograms. (B) The effect of iberin, iberverin and allysin in mixtures (IBR + IBN, ALN + IBR, ALN + IBN). A checkerboard technique was employed to delineate the Fractional Inhibitory Concentration Index (FICI). The treated cultures were screened for visual growth in EnSpire instrument. The FICI values were then calculated according to general method (FICI ≤ 0.5, synergy; 0. 5≤ FICI ≤1.0, additivity; 1.1 ≤ FICI ≤ 2.0, indifference; FICI ≥ 2.0, antagonism)

1. **Analysis of ITC effect against *relA* mutant strain**

**Supplementary Table 1. Antimicrobial activity of ITCs against *relA* strain.** Bacteria were grown in MH medium. Antimicrobial potential is expressed as a minimal inhibitory concentration (MIC).

| **ITC:** | MIC  [mg/l] | MBC  [mg/l] |
| --- | --- | --- |
| **SFN** | 44.3 | 177.2 |
| **SFE** | 43.9 | 87.7 |
| **IBR** | 73.7 | 294.6 |
| **IBN** | 81.7 | 326.6 |
| **ALN** | 191.3 | 382.6 |
| **ERU** | 161.2 | 645.2 |
| **ERY** | 62.5 | 500 |
| **CHE** | 125 | 500 |
